# Retraction Note: ^222^Rn and its relation with meteorological conditions and gaseous pollutants in the outdoor environment of Qena City South of Egypt

**DOI:** 10.1038/s41598-025-28067-6

**Published:** 2025-11-18

**Authors:** Adel. G. E. Abbady, Khaled Salahel Din, Nagwa Saad

**Affiliations:** 1 Physics Department, Faculty of Science, South Valley University, Qena, 83523 Egypt; 2 Faculty of Computer & Information, South Valley University, Qena, 83523 Egypt

Retraction of: *Scientific Reports* 10.1038/s41598-023-45497-2, published online 25 October 2023

The Editors have retracted this Article on the request from the Authors.

After publication, concerns were raised about the radon measurement data reported in this Article. The Authors informed the Journal that this data was collected in 2005 rather than in 2015, as described in the Methodology section. However, the data on meteorological parameters and gaseous pollutants was collected in 2015. The claims of this Article regarding correlations between radon levels, gaseous pollutants, and meteorological parameters therefore no longer stand.

All Authors agree with this retraction and its wording.

